# Evaluation of risk stratification program based on trajectories of functional capacity in patients with acute coronary syndrome: The REACP study

**DOI:** 10.3389/fcvm.2022.1020488

**Published:** 2022-12-20

**Authors:** Dongze Li, Xiaoli Chen, Fanghui Li, Yu Jia, Zhilin Li, Yi Liu, Lei Ye, Yongli Gao, Wei Zhang, Hong Li, Rui Zeng, Zhi Wan, Zhi Zeng, Yu Cao

**Affiliations:** ^1^Department of Emergency Medicine and West China School of Nursing, Laboratory of Emergency Medicine, Disaster Medical Center, West China Hospital, West China School of Medicine, Sichuan University, Chengdu, China; ^2^Department of Cardiology, West China Hospital, West China School of Medicine, Sichuan University, Chengdu, China

**Keywords:** acute coronary syndrome, trajectory, functional capacity, risk stratification, activities of daily living

## Abstract

**Background:**

As a validated assessment tool for functional disability (activities of daily living), the Barthel index (BI) assessed initially at admission has the potential to stratify patients with high-risk acute coronary syndrome (ACS). Dynamic trajectory evaluation of functional capacity in hospitals may provide more prognostic information. We aimed to establish a novel dynamic BI-based risk stratification program (DBRP) during hospitalization to predict outcomes among ACS patients.

**Methods:**

A total of 2,837 ACS patients were included from the Retrospective Multicenter Study for Early Evaluation of Acute Chest Pain. The DBRP rating (low, medium, and high-risk categories) was calculated from dynamic BI at admission and discharge. The primary outcome was all-cause mortality, and the secondary outcome was cardiac mortality.

**Results:**

Of all the included patients, 312 (11%) died during a median follow-up period of 18.0 months. Kaplan–Meier analysis revealed that the cumulative mortality was significantly higher in patients in the higher risk category according to the DBRP. Multivariable Cox regression analysis indicated that, compared to the low-risk category, the higher risk category in the DBRP was an independent strong predictor of all-cause mortality after adjusting for confounding factors (medium-risk category: hazard ratio [HR]: 1.756, 95% confidence interval [95% CI]: 1.214–2.540; *P* = 0.003; high-risk category: HR: 5.052, 95% CI: 3.744–6.817; *P* < 0.001), and the same result was found for cardiac mortality.

**Conclusion:**

The DBRP was a useful risk stratification tool for the early dynamic assessment of patients with ACS.

**Clinical trial registration:**

[http://www.chictr.org.cn], identifier [ChiCTR1900024657].

## 1 Introduction

Acute coronary syndrome (ACS) is a life-threatening emergent condition of coronary artery disease mainly caused by coronary plaque rupture with relatively high mortality and morbidity (1). Risk stratification in patients with ACS facilitates treatment decisions and improves survival rates (1–4). Current guidelines regarding ACS management emphasize the importance of risk assessment for identifying patients with a higher mortality risk requiring more aggressive care and therapy, selecting the optimal care site, and matching therapeutic intensity with risk (1, 5, 6). Previous studies indicated that risk evaluation based on the Global Registry of Acute Coronary Events (GRACE) or thrombolysis in myocardial infarction (TIMI) risk scores have been well-implemented for and proved to be clinically beneficial to patients with ACS, and the ACS guidelines recommend that the GRACE score should be completed within 24 h and re-evaluated before discharge to guide the management of ACS (5–9).

Nearly 38% of in-hospital deaths occur within the first 24 h of symptom onset in patients with AMI; therefore, early, rapid, and dynamic risk assessment identifying high-risk patients is necessary to guide treatment decisions in the emergency department (ED) (10). However, assessment using these risk scores, including GRACE or TIMI, is relatively time consuming and cannot be completed without a medical examination because these scoring systems consist of components including biomarkers of myocardial and other related organ injuries. In addition, the condition of patients with ACS can change rapidly, and the continuous dynamic assessment of ACS patients may provide more prognostic information during the whole course of ACS (11). With this in mind, current scoring systems cannot also immediately stratify patients out of hospitals or during hospitalization, and realize the timely revision of their risk level. This suggests the need for simpler, more accurate dynamic assessment and better treatment decision tools or algorithms to guide individual healthcare during the pre-hospital, admission, in-hospital, and discharge settings.

Activities of daily living (ADL), as a basic functional capacity marker assessed by the Barthel Index (BI) score based on difficulty degrees of daily activities without any laboratory or imaging examination results, has gained interest in recent years as a prognostic indicator in patients with cardiovascular emergency conditions (12, 13). Performance of the ADL assessment is nowadays feasible in the ambulance and, therefore, the functional capacity assessment can be completely obtained in the pre-hospital, in-hospital, or even discharge settings. A previous study indicated that the initial ADL assessed by the BI at the ED has the potential to stratify high-risk patients with ACS, and independently associated with mortality, however, the accuracy was inferior to that of the GRACE score (12). In addition, patients with ACS would receive optimal drug therapy and/or PCI during hospitalization, patient’s ADL should be improved if patients responded well to the treatment therapy, and maybe the elevated change in ADL assessed by BI scores during hospitalization suggested that the improvement of myocardial ischemia or less complications after treatment in hospital. It is possible that the continuous dynamic assessment of functional capacity trajectories may provide more prognostic information for patients with ACS. However, the assessment of functional status at admission or the deterioration in functional status during hospitalization has received little consideration and has not been studied as a potential risk prognostic tool for risk stratification of ACS. Therefore, we conducted this multicenter retrospective cohort study to establish a novel dynamic BI-based risk stratification program (DBRP) based on functional capacity trajectories during hospitalization for long-term outcomes and evaluate the prediction efficiency of this risk assessment tool in patients with ACS.

## 2 Materials and methods

### 2.1 Study design and setting

The Retrospective Evaluation of Acute Chest Pain (REACP) study is a multicenter, retrospective study including a cohort of patients with acute chest pain (ACP) who were admitted to EDs from seven tertiary hospitals in China from January 2017 to December 2019 (clinicaltrials.gov, identifier: ChiCTR1900024657) (12, 14). This study was conducted to elucidate the development of fatal chest pain (ACS, aortic dissection, and pulmonary embolism) and the risk factors in the suspected population. This study was conducted in accordance with the Declaration of Helsinki and approved by local or central institutional review.

### 2.2 Study population

**FIGURE 1:**
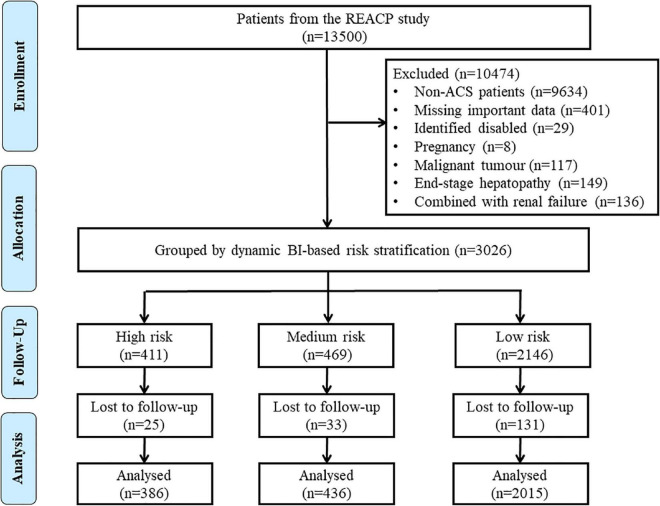
Flow chart of the enrollment of participants in the study. ACS, acute coronary syndrome; BI, Barthel index; REACP, the multicentre retrospective evaluation of acute chest pain study.

In this study, we aimed to establish a novel DBRP based on admission and hospital-acquired BI score for risk stratification in ACS patients. The inclusion criteria were as follows: age greater than 18 years, first-time diagnosis of ST-segment elevation myocardial infarction (STEMI) or non-STEMI (NSTEMI), and unstable angina (UA), less than 12 h between the onset of symptoms and ED admission, and treatment with coronary angiography or primary percutaneous coronary intervention in the hospital. The exclusion criteria were as follows: accompanied by the identified disabled (including previous stroke, severe valvular heart disease, heart failure, chronic obstructive pulmonary disease, rheumatological diseases, trauma diseases, and other diseases with possible impact in BI assessment), malignant tumors, pregnancy, end-stage hepatopathy, or renal failure at admission. A diagram demonstrating the election of patients is shown in Figure 1.

### 2.3 Data collection and measures

In this study, the BI scores of ACS patients were assessed by trained nurses at admission and discharge. The details of evaluating BI scores were described in our previous study (12). Briefly, the BI score comprises 10 items: feeding, toilet use, bathing, grooming, dressing, bowel and bladder control, chair transferring, stair climbing, and ambulating. Each item is scored proportionally, and a given number of points are assigned to each level or rank. The admission and discharge BI assessments were conducted according to responses from the ACS patient or a family member. We divided the BI score into three different level categories according to the standard BI grouping method: high disability caused by ADL (0–40), considered high risk, moderate disability caused by ADL (41–60), considered medium risk, low disability caused by ADL (61–100), considered low risk (12).

We obtained demographic data, characteristic details, and clinical features of the patients from the database of the REACP study, including medical histories, vital signs, electrocardiograms, troponin I/T, myocardial enzymes, liver and renal function, coronary angiography (CAG) findings, echocardiography findings, inpatient complications, pre-hospital and in-hospital treatment and discharge medication. Standard case report forms were used to collect these data; the details were described in our previous publications (3, 4, 12, 15, 16).

### 2.4 Risk stratification score based on dynamic BI scores

**FIGURE 2:**
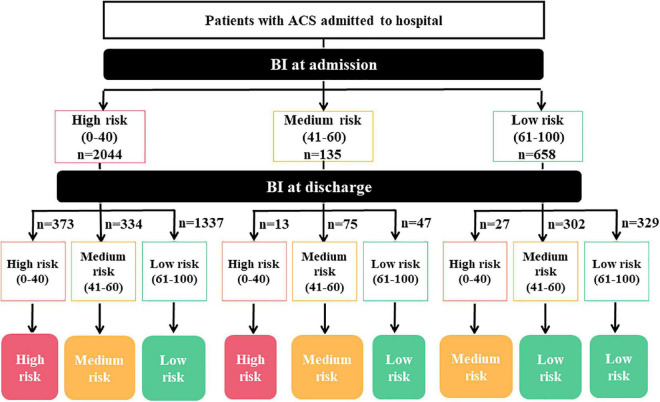
Diagram of the detailed rules for dynamic BI-based risk stratification. BI, Barthel index.

In this study, we established a novel DBRP consisting of the low, medium, and high-risk categories, based on admission BI, discharge BI and the changes between the two. In terms of the BI changes, “largely improved” was defined as two levels of improvement; for example, BI changes from the high risk (0–40) to the low-risk category (61–100); “slightly improved” was defined as one level of improvement; for example, BI changes from the high-risk category (0–40) to the medium-risk category (41–60); “largely declined” was defined as two levels of worsening; for example, BI changes from low risk (61–100) to high risk (0–40); “slightly declined” was defined as one level of worsening; for example, BI changes from the medium risk (41–60) to the high-risk category (0–40); “no change” was defined as the risk group at discharge BI remaining the same as that on admission. In particular, for dead patients within hospitalization, the BI at discharge was signed to high risk (0–40). The detailed rules for risk stratification based on the DBRP are described in Figure 2.

### 2.5 Outcome and follow-up

The primary endpoint of this study was all-cause mortality, confirmed through a combination of hospital medical records and telephone contact with the patient’s family members. The secondary outcome was cardiac death, identified based on hospital record reviews for identified hospitalizations and through phone interviews. All reported events were reviewed and verified by the outcome assessment committee of the REACP study.

### 2.6 Statistical analysis

Parametric continuous variables are expressed as means ± standard deviations (SD) and non-parametric continuous variables as medians with interquartile ranges. Categorical variables are reported as frequencies and percentages. Parametric patient characteristics were compared using one-way analysis and non-parametric variables using the Kruskal–Wallis H test. Categorical variables were compared using Fisher’s exact test or chi-square test.

The Kaplan–Meier survival analysis and log-rank tests were performed to calculate and compare the cumulative survival of ACS patients with different risk levels. Cox proportional hazards models were used to investigate the relationship between risk levels according to dynamic BI-based risk stratification and time-to-mortality. Hazard ratios (HRs) and 95% confidence intervals (CIs) were calculated using the multivariate Cox regression model after adjusting for potential influencing factors. The sensitivity, specificity, accuracy, positive predictive value (PPV), negative predictive value (NPV), and Cohen’s Kappa coefficient were calculated to evaluate the predictive efficiency of DBRP and GRACE score. Receiver operating characteristic (ROC) analyses for the DBRP and GRACE score were performed, and differences in mortality between these indicators were compared using the area under the curve (AUC) values with the method of DeLong et al. (17).

Subgroup analysis was performed to test the robustness of the association between the Dynamic BI-based risk stratification score and the all-cause mortality. A two-tailed *P*-value < 0.05 was considered significant for all tests. Statistical analyses were performed using SPSS version 22.0 (IBM Corp, Armonk, NY, USA) and R Statistical Software (v4.1.2; R Core Team 2021) (18).

## 3 Results

### 3.1 Baseline patient characteristics

A total of 2,837 ACS patients were enrolled with an average age of 65.5 ± 13.0 years. Of these participants, 2,121 (74.7%) were male. According to the dynamic BI-based risk stratification program (DBRP), patients were divided into three groups: the high-risk (*n* = 386, 13.6%), medium risk (*n* = 436, 15.4%), and low-risk groups (*n* = 2,015, 71.0%). During a median follow-up period of 18.0 (10.3–24.2) months, a total of 312 (11.0%) patients died, of whom 237 (8.3%) died due to cardiac causes. The baseline characteristics of patients in these three groups are described and compared in Table 1. Compared to those in the low-risk group, participants in the high-risk group were older, had lower body mass indexes (BMI), systolic blood pressure (SBP), diastolic blood pressure (DBP), left ventricular ejection fraction (LVEF), triglycerides, total cholesterol, and low-density lipoprotein (LDL), and had higher heart rates, fibrinogen, blood glucose, creatinine, BUN, N-terminal pro-B-type natriuretic peptide (NT-proBNP), cardiac troponin T, creatinine kinase, CK-MB levels, GRACE scores, and Genisini scores, and were more likely to have chronic obstructive pulmonary disease (COPD) and Killip classes ≥ 2. Several common inflammatory and thrombus indicators, namely white blood cells (WBC), neutrophil, C-reactive protein (CRP), interleukin 6, neutrophil-to-lymphocyte ratio (NRL), and D-dimer, were significantly higher in the high-risk category of DBRP than in the low-risk category (NLR, *P* = 0.009; all others, *P* < 0.001).

**TABLE 1:** Relationships between baseline clinical characteristics and the DBRP in patients with acute coronary syndrome.

Characteristic	High-risk (*n* = 386)	Medium-risk (*n* = 436)	Low-risk (*n* = 2,015)	*P*-value
**Demographic variables**				
Age, years	70.5 ± 13.9	69.6 ± 13.1	63.6 ± 12.4	<0.001
Males, n (%)	264 (68.4%)	288 (66.1%)	1569 (77.9%)	<0.001
Smoking, n (%)	181 (46.9%)	204 (46.8%)	1162 (57.7%)	<0.001
Drinking, n (%)	89 (23.1%)	114 (26.3%)	695 (34.5%)	<0.001
**Chronic medical conditions**				
Hypertension, n (%)	210 (54.4%)	264 (60.6%)	1055 (52.4%)	0.008
Diabetes, n (%)	126 (32.6%)	147 (33.7%)	489 (24.3%)	<0.001
Hyperlipidemia, n (%)	37 (9.6%)	53 (12.2%)	253 (12.6%)	0.260
COPD, n (%)	20 (5.2%)	19 (4.4%)	45 (2.2%)	0.001
**Physiological and lab variables**				
BMI, kg/m^2^	23.4 ± 3.6	23.9 ± 3.6	24.4 ± 3.2	<0.001
Admission SBP, mmHg	123 ± 25.8	131.2 ± 25.3	129.4 ± 23.4	<0.001
Admission DBP, mmHg	74.8 ± 17.1	77.4 ± 15.6	79.5 ± 15.4	<0.001
Heart rate, /min	87.0 ± 22.8	81.9 ± 18.8	79.4 ± 16.8	<0.001
Killip class ≥ 2, n (%)	233 (60.4%)	223 (51.1%)	770 (38.2%)	<0.001
LVEF, (%)	49.0 ± 13.3	53.1 ± 12.1	55.4 ± 11.2	<0.001
WBC, 10^9^/L	10.6 ± 4.3	9.6 ± 3.6	9.2 ± 3.5	<0.001
Neutrophil, 10^9^/L	7.9 ± 3.9	7.7 ± 3.6	7.3 ± 3.5	<0.001
CRP, mg/L	46.5 (15.9–90.2)	5.1 (2.8–12.9)	6.6 (2.7–33.2)	<0.001
IL-6, pg/mL	28.3 (13.3–52.8)	9.5 (5.4–26.3)	12.6 (6.4–43.4)	<0.001
NLR	5.5 (2.9–10.1)	5.5 (3.2–9.0)	5.0 (3.0–8.4)	0.009
Platelet count, 10^9^/L	170.5 (135–213)	174 (138–223)	177 (140–219)	0.755
D-dimer, mg/L	0.8 (0.4–1.9)	0.6 (0.3–1.1)	0.3 (0.2–0.7)	<0.001
Fibrinogen, g/L	3.4 (2.7–4.6)	3.3 (2.7–4.3)	2.9 (2.4–3.7)	<0.001
Blood glucose, mmol/L	8.1 (6.5–11.2)	7.8 (6.4–10.4)	7.4 (6.1–9.7)	<0.001
Creatinine, μmol/L	90 (71.5–130.5)	82 (69–106)	77 (65–91)	
BUN, mmol/L	7.1 (5.3–10.3)	6.2 (5–8.6)	5.6 (4.5–7)	<0.001
Triglycerides, mmol/L	1.2 (0.8–1.6)	1.3 (0.9–2.1)	1.5 (0.9–2.2)	<0.001
Total cholesterol, mmol/L	4.2 ± 1.2	4.3 ± 1.3	4.5 ± 1.3	<0.001
HDL, mmol/L	1.2 ± 0.4	1.1 ± 0.4	1.1 ± 0.3	0.006
LDL, mmol/L	2.6 ± 1.1	2.7 ± 1.1	2.8 ± 1.1	0.001
NT-proBNP, pg/mL	2399 (382–6316)	1490 (404–4221)	499 (138–1594)	<0.001
CTn T pg/mL	1020 (191–4322)	387 (60–1872)	301 (31–1517)	<0.001
Creatinine kinase, IU/L	290 (126–1150)	155 (72–627)	172 (87–675)	0.008
CK-MB, U/L	14.3 (4.1–76.9)	7.4 (2.5–32.4)	6.5 (2.1–52.1)	0.083
Stenotic coronary arteries*				
Left main, n (%)	75/374 (20.1%)	94/426 (22.1%)	330/1985 (16.6%)	0.015
LAD, n (%)	249/374 (66.6%)	358/426 (84.0%)	1737/1985 (87.5%)	<0.001
Left circumflex, n (%)	215/374 (57.5%)	299/426 (70.2%)	1305/1985 (65.7%)	0.001
RCA, n (%)	230/374 (61.5%)	331/426 (77.7%)	1546/1985 (77.9%)	<0.001
**Risk score**				
GRACE score	170.0 ± 45.7	153.2 ± 37.6	139.2 ± 35.7	<0.001
Gensini score*	84 (43–120)	67 (37–107)	56 (29–90)	<0.001
Treatment				0.002
PCI, n (%)	267 (69.2%)	348 (79.8%)	1521 (75.5%)	
Optimal drug therapy, n (%)	119 (30.8%)	88 (20.2%)	494 (24.5%)	

SBP, systolic blood pressure; DBRP, dynamic Barthel index-based risk stratification program; DBP, diastolic blood pressure; BMI, body mass index; COPD, chronic obstructive pulmonary disease; WBC, white blood cell count; BUN, blood urea nitrogen; HDL, high-density lipoprotein; LDL, low-density lipoprotein; CTn T, cardiac troponin T; NT-proBNP, N-terminal pro-brain natriuretic peptide; CK-MB, creatinine kinase-myocardial band isoenzyme; CRP, C-reactive protein; LVEF, left ventricular ejection fraction; LAD, left anterior descending; NLR, neutrophil-to-lymphocyte ratio; RCA, right coronary artery; GRACE, the Global Registry of Acute Coronary Events score; PCI, percutaneous coronary intervention.

*Two thousand seven hundred and eighty-five patients received coronary angiography, and other 52 patients refuse to undergo coronary angiography.

### 3.2 The dynamic BI-based risk stratification program and clinical outcomes

**FIGURE 3:**
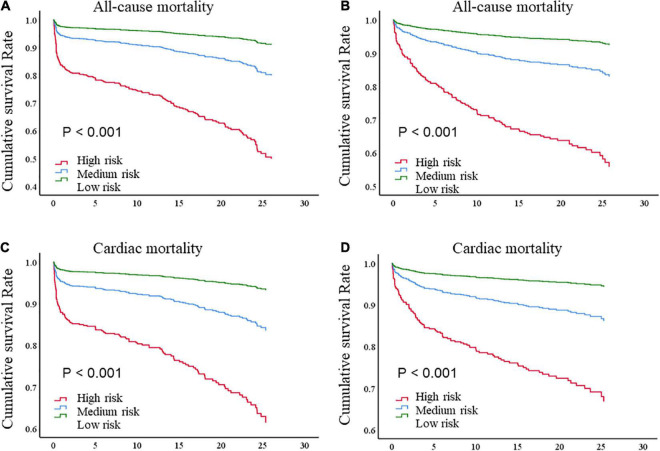
Kaplan–Meier survival curve of **(A)** all-cause death for STEMI patients; **(B)** all-cause death for NST-ACS patients; **(C)** cardiac death for STEMI patients; **(D)** cardiac death for NST-ACS patients; by risk levels according to dynamic BI-based risk stratification. BI, Barthel index; STEMI, ST-segment elevation myocardial infarction; NST-ACS, non-ST elevation acute coronary syndrome.

Kaplan–Meier analysis (Figure 3) revealed that the cumulative mortality was significantly higher in patients in the higher risk category according to the DBRP, regardless of STEMI and non-ST segment elevation acute coronary syndrome (NST-ACS), in both all-cause mortality and cardiac mortality (*P* < 0.001 for all). Multivariable Cox regression analysis further indicated compared to participants with the low-risk category, the higher risk category in the DBRP was an independent strong predictor of both all-cause mortality and cardiac mortality after eliminating confounding factors (all-cause mortality: medium-risk category: HR: 1.756, 95% CI: 1.214–2.540; *P* = 0.003; high-risk category: HR: 5.052, 95% CI: 3.744–6.817; *P* < 0.001; cardiac mortality: medium-risk category: HR: 1.865, 95% CI: 1.252–2.779; *P* = 0.002; high-risk category: HR: 4.780, 95% CI: 3.423–6.673; *P* < 0.001; Table 2).

**TABLE 2:** Cox regression analysis regarding correlations between clinical outcomes and the DBRP.

Variables	Unadjusted		Model 1		Model 2		Model 3	
	HR (95% CI)	*P*-value	HR (95% CI)	*P*-value	HR (95% CI)	*P*-value	HR (95% CI)	*P*-value
**All-cause mortality**								
Low-risk	REF.	–	REF.	–	REF.	–	REF.	–
Medium-risk	2.352 (1.721–3.215)	<0.001	1.891 (1.350–2.650)	<0.001	1.959 (1.390–2.759)	<0.001	1.756 (1.214–2.540)	0.003
High-risk	7.499 (5.853–9.606)	<0.001	5.086 (3.842–6.733)	<0.001	5.079 (3.818–6.757)	<0.001	5.052 (3.744–6.817)	<0.001
**Cardiac mortality**								
Low-risk	REF.	–	REF.	–	REF.	–	REF.	–
Medium-risk	2.536 (1.791–3.592)	<0.001	2.055 (1.427–2.959)	<0.001	2.095 (1.446–3.034)	<0.001	1.865 (1.252–2.779)	0.002
High-risk	7.056 (5.296–9.402)	<0.001	4.865 (3.556–6.656)	<0.001	4.851 (3.529–6.668)	<0.001	4.780 (3.423–6.673)	<0.001

Model 1: adjusted by age, sex, admission systolic blood pressure, smoking, drinking, body mass index, hypertension, diabetes.

Model 2: adjusted by model 1 plus white blood cell count, creatinine kinase-myocardial band isoenzyme, cardiac troponin T, blood urea nitrogen.

Model 3: adjusted by model 2 plus Global Registry of Acute Coronary Events score and Gensini score. CI, confidence interval; DBRP, dynamic Barthel index-based risk stratification program; HR, hazard ratio; REF, reference.

### 3.3 The dynamic BI-based risk stratification program and its predictive efficiency

The sensitivity, specificity, accuracy, PPV, and NPV for mortality of the dynamic BI-based risk stratification, when high-risk was taken as the cut point, were 42.0, 89.9, 84.6, 33.9, and 92.6%, respectively. And the Cohen’s Kappa coefficient was 0.289 (95%CI: 0.240–0.338, *P* < 0.001). When medium-risk was used as the cutoff point, the sensitivity, specificity, accuracy, PPV, and NPV for mortality of the dynamic BI-based risk stratification were 60.6, 74.9, 73.4, 23.0, and 93.9%, respectively. The Cohen’s Kappa coefficient was 0.207 (95% CI: 0.171–0.242, *P* < 0.001). We also evaluated the predictive efficiency of the GRACE score, using guideline-recommended 140 and 108 as cutoff points, respectively. The specific results were shown in Table 3. The AUC generated using the ROC curve analysis found no significant differences in AUCs for all-cause mortality between the DBRP (low, medium, and high risk) and GRACE score (140 and 108 as cutoff points) (AUC, 0.700 vs. 0.698, *P* > 0.05), however, the AUC of GRACE score (AUC, 0.791, *P* < 0.001) was higher than that of categorical DBRP for all-cause mortality.

**TABLE 3:** Predictive efficiency for mortality of the DBRP and GRACE score in acute coronary syndrome patients.

Values	Sensitivity (%)	Specificity (%)	Accuracy (%)	PPV (%)	NPV (%)	Kappa (95% CI)
**DBRP**						
High risk	42.0	89.9	84.6	33.9	92.6	0.289 (0.240–0.338)
Medium risk	60.6	74.9	73.4	23.0	93.9	0.207 (0.171–0.242)
**GRACE score**						
140	85.9	52.5	56.2	18.3	96.8	0.146 (0.124–0.168)
108	97.1	18.8	27.4	12.9	98.1	0.041 (0.033–0.049)

AUC, area under the curve; DBRP, dynamic Barthel index-based risk stratification program; PPV, positive predictive value; NPV, negative predictive value; BI, Barthel index; CI, confidence interval; GRACE, the Global Registry of Acute Coronary Events.

### 3.4 Subgroup analysis

We carried out subgroup analysis by grouping patients according to gender, age, BMI, SBP, DBP, heart rate, WBC, cardiac troponin T, NT-proBNP, Killip class, GRACE score, and ACS type. Patients in the high-risk group had the lowest cumulative survival rates of all-cause mortality in each subgroup (Table 4).

**TABLE 4:** Kaplan–Meier survival analysis of mortality in acute coronary syndrome patients.

Subgroups	Cumulative survival rate			Log rank χ^2^	*P*-value
	High-risk	Medium-risk	Low-risk		
**Gender**					
Male (*n* = 2,121)	0.488	0.823	0.937	293.382	<0.001
Female (*n* = 716)	0.497	0.838	0.846	55.749	<0.001
>**Age^a^**					
≤65 (*n* = 1,345)	0.794	0.923	0.967	79.083	<0.001
>65 (*n* = 1,492)	0.347	0.786	0.859	187.264	<0.001
>**BMI,^b^ kg/m^2^**					
≤24 (*n* = 1,508)	0.365	0.763	0.889	186.051	<0.001
>24 (*n* = 1,329)	0.700	0.891	0.942	88.698	<0.001
>**SBP,^b^ mmHg**					
≤128 (*n* = 1,455)	0.432	0.815	0.916	212.282	<0.001
>128 (*n* = 1,382)	0.593	0.840	0.920	116.962	<0.001
>**DBP,^b^ mmHg**					
≤78 (*n* = 1,486)	0.440	0.845	0.907	172.766	<0.001
>78 (*n* = 1,351)	0.563	0.807	0.930	166.130	<0.001
>**Heart rate,^b^ /min**					
≤ 78 (*n* = 1,444)	0.555	0.867	0.934	103.610	<0.001
>78 (*n* = 1,393)	0.449	0.794	0.901	211.153	<0.001
>**WBC,^b^ 10^9^/L**					
≤9 (*n* = 1,477)	0.639	0.860	0.927	103.938	<0.001
>9 (*n* = 1,360)	0.412	0.795	0.907	219.677	<0.001
>**Troponin T,^b^ pg/mL**					
≤453 (*n* = 1,438)	0.710	0.850	0.930	65.640	<0.001
>453 (*n* = 1,399)	0.337	0.809	0.902	224.779	<0.001
>**NT-proBNP,^b^ pg/mL**					
≤745 (*n* = 1,452)	0.872	0.949	0.956	24.895	<0.001
>745 (*n* = 1,385)	0.293	0.766	0.865	196.706	<0.001
>**Killip class^c^**					
I (*n* = 1,611)	0.720	0.894	0.945	56.387	<0.001
II-IV (*n* = 1,226)	0.330	0.768	0.874	204.638	<0.001
>**GRACE score^b^**					
≤142 (*n* = 1,443)	0.891	0.900	0.973	28.693	<0.001
>142 (*n* = 1,394)	0.369	0.781	0.856	205.622	<0.001
>**ACS type**					
STEMI (*n* = 1,581)	0.452	0.824	0.921	252.327	<0.001
NST-ACS (*n* = 1,256)	0.327	0.832	0.915	95.472	<0.001

HR, hazard ratio; CI, confidence interval; BMI, body mass index; SBP, systolic blood pressure; DBP, diastolic blood pressure; WBC, white blood cell; GRACE score, Global Registry of Acute Coronary Events score; ACS, acute coronary syndrome; STEMI, ST-elevation myocardial infarction; NST-ACS, non-ST elevation acute coronary syndrome.

^a^The cutoff point for age was according to the definition of the elderly (65 years old).

^b^The cutoff points for these variates were medians.

^c^The cutoff point for Killip class was having congestive heart failure (≥II).

## 4 Discussion

This study established a novel dynamic BI-based risk stratification program (DBRP) using admission and discharge BI, and the changes between them for risk assessment in ACS patients and investigated whether the DBRP was efficient in predicting the prognosis of patients with ACS. Our findings demonstrated that the DBRP could accurately predict the prognosis of ACS patients. Patients with high and medium risks were correlated with an increased risk of all-cause mortality and cardiac mortality compared to those with low risk. Higher risk independently predicted a worse prognosis in ACS patients.

Our previous study demonstrated that the BI scores assessed at admission were a valuable prognostic predictor for patients with ACS, predicting all-cause mortality and cardiac mortality both in-hospital and during follow-up (12). According to the BI scores at admission, the HR for mortality of patients in the high-risk group is twice that of patients in the low-risk group. One study focusing on older ACS patients (≥85 years) found that the BI scores assessed at discharge were correlated with 1-year mortality in these patients (13). However, the development of the disease process is ever-changing; thus, dynamic assessment may provide more valuable information (11).

The BI score at admission reflects the ADL of patients before medical intervention, which shows the initial status of the patient after the onset of illness, while the BI score assessed at discharge reflects the ADL of patients after receiving medical intervention, indicating the patient’s current status. The change between these two indices provided information on disease development and therapeutic effects. The DBRP comprehensively evaluates these three items, which may more accurately predict the prognosis of ACS patients. In addition, the elevated change in ADL assessed by BI scores during hospitalization suggested that the improvement of myocardial ischemia or less complications after treatment in hospital. Therefore, In this study, we took the dynamic changes of ADL functional status into consideration and established a relatively more accurate risk stratification tool for ACS patients.

As described in our previous research, ADL representing patients’ physical functional status is correlated with several pathophysiological states, including inflammatory processes, aging status, and frailty (19–23). These factors are all essential considerations in the occurrence and development of cardiovascular disease (24–28). Also, the results of our study showed that several common inflammatory and thrombus indicators were significantly higher among patients in the high-risk category of DBRP than in the low-risk category, which may further explain an underlying mechanism. Prior studies have demonstrated that the indicators related to the various pathophysiological conditions involved in the pathogenesis of cardiovascular disease or myocardial injury may provide more prognostic information (3, 29–31). Being a validated evaluation tool for ADL, the BI score is highly likely to play a role in the risk stratification and prognosis prediction of ACS patients.

As the performance of the evaluation of ADL is nowadays feasible in the ambulance, the BI score can be completely obtained in the pre-hospital setting. The scale is considered easy to use, with good reliability and sensitivity to change, mainly in predicting the ADL functional status. The DBRP established in the present study is based on the changes between admission and discharge BI scores, and it has been proved that dynamic monitoring may provide more information and guide clinical decision-making, no matter the patient’s physiological indices or functional status (32–35). Thus, the continuous dynamic evaluation of BI scores may provide more prognostic information for patients with ACS. According to our results, the risk of mortality was five times greater in the high-risk category than the low-risk category of DBRP. Furthermore, in patients with different levels of cardiovascular risk factors, the DBRP had a stable prognostic value. This result is far better than that of our previous study, in which risk stratification was carried out based on the BI score at admission alone (12).

The GRACE score is a guideline-recommended risk stratification for patients with ACS, comprising several factors, including demographic data, heart and other organ damage related to ACS, and has been widely used in clinical practice (36). The results of this study show that the DBRP had relatively better specificity, accuracy, PPV, and consistency than the GRACE score in predicting mortality in ACS patients, and no significant differences in the AUCs for all-cause mortality were observed between the DBRP and GRACE score (as categorical data). The DBRP provides additional geriatric-related signals reported to predict outcomes beyond age and standard risk factors (37). Currently, the ACS patients with geriatric conditions account for an increasing proportion of total patients, making it all the more important to consider the relevant indicators (38). Thus, the DBRP is indeed necessary because it may provide prognostic information not provided by the GRACE score, and combining these two indicators may illustrate comprehensive and systematic information. Furthermore, the BI score is routinely evaluated orally by nurses in hospital settings in China, which does not increase the burden on doctors, and has been widely accepted by both physicians and patients. Importantly, in the early evaluation of ACS patients, the participation of nurses can promote physician-nurse collaboration, subsequently leading to a more efficient and comprehensive evaluation. The latest European Society of Cardiology consensus statement demonstrated that the active participation of well-trained nurses can be beneficial to the risk stratification of patients (5). In addition, as a ADL assessment tool, the BI score consists of 10 items that relate to ADL without any medical examination results, and is considered easy to use mainly in predicting the functional outcomes.

## 5 Limitations

There are several limitations to this study. Firstly, retrospective as this study was, large, multicenter, and prospective studies are needed to further verify the validity of these results. Secondly, we only collected BI scores at one time point after admission, while multiple collections may provide more prognostic information. Thirdly, whether subsequent clinical interventions according to the DBRP can improve the prognosis of ACS patients was not investigated in this study, and this would be an interesting point to further explore in the future.

## 6 Conclusion

This study established a risk stratification tool based on dynamic BI scores and demonstrated that this dynamic BI-based risk stratification program might help identify high-risk patients and provide useful prognostic information for patients with ACS. As such, it could be applied in clinical practice for ACS patients for early risk warning and clinical decision guidance.

## Data availability statement

The original contributions presented in this study are included in this article/supplementary material, further inquiries can be directed to the corresponding authors.

## Author contributions

DL, ZZ, and YC conceived the study design. DL, XC, YJ, FL, ZL, YL, WZ, LY, HL, and YG collected the epidemiological and clinical data. DL, XC, YJ, YL, and ZL summarized data and performed the statistical analysis. DL, FL, and YC interpreted the data and drafted the manuscript. ZZ and RZ participated in the design of the study, acquired the data, and helped to revise the manuscript. All authors accepted responsibility for the entire content of this submitted manuscript and approved submission.
